# Optimizing alcohol screening according to DSM-5 severity by adaptive testing using the AUDIT

**DOI:** 10.1186/1940-0640-10-S2-O8

**Published:** 2015-09-24

**Authors:** Gallus Bischof, Anja Bischof, Christian Meyer, Hans-Juergen Rumpf

**Affiliations:** 1 Dpt. of Psychiatry, University of Luebeck, Luebeck, Germany; 2 Institute of Epidemiology and Social Medicine, University of Greifswald, Greifswald, Germany

## Background

Due to unsatisfactory results of alcohol-related SBI in patients with severe Alcohol Use Disorders (AUDs), many studies aim to exclude severe cases by defining a cut-off in the upper range of the Alcohol Use Disorders Identification Test AUDIT. However, research focusing on optimal cut-offs is still insufficient. In addition, cut-off values of the AUDIT for DSM-5 substance use disorder criteria have not been analysed so far.

## Material and methods

Data were collected in a general population sample (n=4.075). Alcohol consumption and alcohol use disorders were assessed using the Munich Composite International Diagnostic Inventory (M-CIDI) in all participants as the gold standard. Participants were then grouped according to M-CIDI into individuals without alcohol-related risks, at-risk consumers without AUDs and individuals with alcohol use disorders according to DSM-5. Alcohol Use data was dichotomized into belonging to the target group of SBI (at-risk drinking and/or mild or moderate AUD) vs. not belonging to the target group (unrisky drinking pattern or severe AUD).

## Results

In both samples, the best inclusion criteria on the lower end was a cut-off value of 5 points in the AUDIT-C. Combining AUDIT-C with 5 points and the remaining AUDIT-items with a cut-off of maximum 6 points in order to exclude severe AUDs yielded the best performance. The Area under the Curve (AUC) for adaptive testing using these cut-off values was significantly better than using a simple AUDIT in-/exclusion rule of 5 and 11 points (AUC .77 vs. .73, p=.01).

## Conclusions

Data suggest that adaptive screening can improve the identification of individuals with at risk drinking and non-severe AUDs. Further research on the performance of adaptive screening in trials on SBI is warranted.

